# High-Precision Electrosurgical Scalpel for Scarpa Sparing Abdominoplasty – Pilot Randomized Controlled Trial of Efficacy and Safety

**DOI:** 10.1007/s00266-026-05621-9

**Published:** 2026-01-26

**Authors:** Sara Castro-Pinto, Gonçalo Gandra, António de Sousa Barros, Marco Rebelo, Helena Peres, António Costa-Ferreira

**Affiliations:** 1 Departement of Surgery and Physiology, Faculty of Medicine, Porto University, Porto, Portugal; 2 Department of Plastic, Reconstructive and Aesthetic Surgery, São João University Hospital, Porto, Portugal; 3 Santa Casa da Misericórdia de Lousada Hospital, Lousada, Portugal; 4 Department of Biology, Faculty of Sciences, Porto University, Porto, Portugal; 5 Plastic Surgery Department, Portuguese Institute of Oncology, Porto, Portugal

**Keywords:** Abdominoplasty, Scarpa fascia preservation, Body contour, Electrocautery, High-precision electrocautery tip, Low heat conduction

## Abstract

**Background:**

The dissection technique can influence abdominoplasty complication rates. New “bovie” tips have been developed to minimize thermal damage and optimize tissue dissection.

**Objectives:**

The purpose of the present study was to perform a randomized controlled trial to evaluate the effect of the dissection technique on several outcomes and complications after a Scarpa-sparing abdominoplasty, comparing a high-precision electrosurgical scalpel (HPES) with the conventional electroscalpel.

**Methods:**

This prospective study was conducted at a single medical facility between June 2020 and February 2022 and enrolled patients who underwent Scarpa-sparing abdominoplasty. Forty female patients were included in the study and randomly assigned to either abdominoplasty performed using the conventional “bovie” tip (Group A) or a similar procedure using the HPES tip (Group B). The following variables were analyzed: patient characteristics, duration of suction drain use, drain output, complications (local and systemic), unscheduled visits, readmissions, and reoperation requirements.

**Results:**

Both groups exhibited similar general characteristics, differing only in body mass index. The HPES group showed a 23.6% significant reduction in total drain output and a trend toward lower daily outputs, peaking on day two with a 60.0% reduction in drain output. Local and systemic complications were similar, except for a trend toward lower wound dehiscence in the HPES group. Notably, no systemic complications occurred in either group.

**Conclusion:**

Our findings support the safety and efficacy of the HPES tip for Scarpa-sparing abdominoplasty. This pilot randomized controlled trial demonstrated the benefits of substantially reducing drain output.

**Level of Evidence II:**

This journal requires that authors assign a level of evidence to each article. For a full description of these Evidence-Based Medicine ratings, please refer to the Table of Contents or the online Instructions to Authors www.springer.com/00266.

## Introduction

Abdominoplasty is a widely performed surgery, and it is crucial to achieve optimal results with minimal complications. A meta-analysis of 15 studies on abdominoplasty reported an average 39% complication rate and 23% seroma rate [1]. Several strategies have been reported to reduce the complication rate, such as progressive tension sutures [2–4], fibrin glue [5], Scarpa fascia preservation [6–9], lipoabdominoplasty [10], selective undermining [11], and closed-suction drains [12–14].

Nevertheless, the dissection method has also been implicated in complication rates. Previous studies have compared different existing devices for raising the abdominal flap, from steel scalpels to several energy-based systems [15–21]. However, their definitive superiority needs to be established [20]. Dissection performed using a steel scalpel provides a cleaner cut [18]. It has shown advantages in the postoperative period, specifically regarding lower drain outputs and seroma rates [17, 18]. In contrast, electrosurgery allows tissue cutting while simultaneously performing hemostasis [21]. However, inducing thermal damage to the surrounding tissues [20] may lead to a higher formation of seromas [22].

The etiology of seromas is not fully understood. Still, a plausible link exists between thermal tissue damage, subsequent inflammatory exudate [23], and the concomitant involvement of lymphatic vessels, resulting in increased formation of fluid collections [24].

**Fig. 1:**
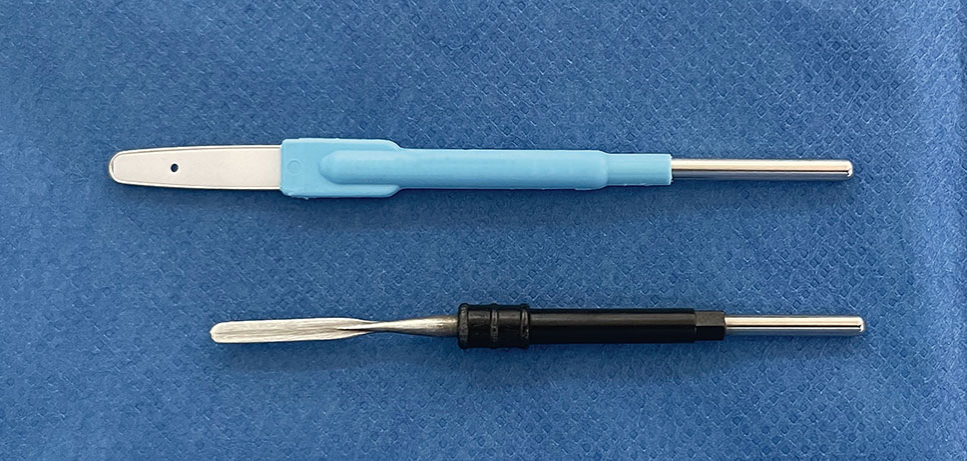
Electroscalpels used in full abdominoplasty with Scarpa fascia preservation. From top to bottom: the HPES (high-precision electrosurgical scalpel) from Utah Medical Products EPITOME® 0.2 Disposable Electrosurgical Electrode (Midvale, UT); the traditional “bovie” tip (ERBE VIO 300S (Erbe Elektromedizin GmbH, Tuebingen, Germany)

New “bovie” tips have been recently developed to reduce the thermal effects of conventional electrosurgery through improved design, enhanced precision, and poor heat-conducting materials [25]. A high-precision electrosurgical scalpel (HPES) comprises a peripheral conductive thin wire with a nonconductive central ceramic core (Fig. 1). This electrode tip is shaped to facilitate soft tissue dissection due to the extensive contact area, wider than the regular “bovie” tip, while at the same time minimizing heat transfer during tissue dissection due to the nonconductive ceramic core [25]. This pilot study aimed to evaluate the impact of the dissection method on abdominoplasty with Scarpa fascia preservation by comparing an HPES tip with a conventional tip.

## Methods

This prospective study was conducted at a single medical facility between June 2020 and February 2022. It included patients who underwent a full abdominoplasty using a Scarpa-sparing technique. The institution’s Ethical Committee approved this study, and all participating patients provided Informed Consent. The study was compiled according to the Consolidated Standards of Reporting Trials Group statement (CONSORT).

Forty consecutive female patients meeting the criteria for full abdominoplasty with umbilical transposition (Bozola types III/IV and Matarasso types III/IV) [26, 27] were selected from the outpatient clinic. Exclusion criteria included significant medical comorbidities, bariatric patients without weight stabilization for at least 6 months, patients who anticipated future pregnancy, and those with a body mass index(BMI) ≥ 30kg/m^2^ unless having a previous bariatric surgery.

The same fully trained surgeon performed all the surgical procedures. A total of 40 patients were enrolled in this study and were assigned randomly to have the flap dissection done with the traditional “bovie” tip (group A) or the HPES® Electrode (group B). A random computer-generated list was used to allocate participants, ensuring an unbiased distribution across the two groups.

### Surgical Technique

The patients were administered preoperative enoxaparin (40 mg/day subcutaneously during the hospital stay, a 2-hour minimal interval before surgery) and prophylactic broad-spectrum intravenous antibiotics. All patients underwent full abdominoplasty with preservation of Scarpa fascia and the deep fat compartment in the infraumbilical area. The abdominal flap was dissected using two different planes: on top of the Scarpa fascia in the lower abdomen and on top of the deep fascia in the epigastric region and infra umbilical midline. The procedures included umbilical transposition and muscle plication. The surgical technique, including preoperative markings, has been comprehensively described elsewhere [1, 6, 28].

The diathermocoagulation device employed was the ERBE VIO 300S (Erbe Elektromedizin GmbH, Tuebingen, Germany) with coagulation set to forced coagulation, effect 4, coagulation regulated to 50 Watts, and spray mode deactivated. These settings were consistent for all the procedures. In group A, patients were treated with a conventional tip (Erbe Elektromedizin GmbH, Tuebingen, Germany). In contrast, in group B, patients underwent dissection with the HPES EPITOME® electrode (Utah Medical Products Epitome 0.2 Disposable Electrosurgical Electrode (Midvale,UT)). Electrocautery in the coagulation mode was used for all tissue dissections in both groups. The cut mode was not used in this study.

Liposuction was limited to the flanks and did not involve the upper abdominal flap. Compression garments were used in all patients and applied in the operating room immediately after the surgery. No additional abdominal procedure was done. Two closed-suction drains on the lower abdomen were used in all patients, one on each side. Drains were removed when the patients were ambulatory, and the daily drain output on each drain was 30 mL or less. The patients were encouraged to ambulate on the first postoperative day and instructed to avoid strenuous activity and use compression garments for at least 6 weeks.

### Outcomes

The study assessed various outcomes, including the duration of suction drain use, drain output(total and daily), local and systemic complications, unscheduled appointments, new admission to the hospital, and secondary surgical procedures. Local complications included hematoma, seroma, wound rupture, and wound infection. Systemic complications included deep vein thrombosis(DVT), pulmonary thromboembolism, and death. The drain output was recorded daily by a nurse unaware of the type of device used for flap dissection.

### Statistical Analysis

The statistical analyses were performed using R (version 4.3.2) and *gtsummary* R package [29, 30]. Descriptive statistics were computed for all variables as appropriate. We used the *χ*^2^ (Chi-squared) test for categorical data, and Fisher’s exact test was used in cases where the expected frequencies were too small for the *χ*^2^ test. A probability level of 0.05 was used to reject the null hypothesis.

Regression analysis was applied to establish the associations between drain volume and variables such as age, BMI, scalpel type, and drain use duration. The model was constructed with drain volume as the dependent variable, whereas age, BMI, scalpel type, and number of days with drains were treated as independent variables. Regression coefficients (beta) provided estimates of the relationships between these variables and drain volume. In addition, marginal effects were computed to assess the average impact of the scalpel type on the drain volume. The marginal effects measure the change in the expected drain volume for a one-unit change in the continuous predictors and scalpel type, holding all other variables constant. Given the pilot nature of this study and its limited sample size, bootstrap resampling with 1000 iterations was performed to obtain more robust estimates of the covariates’ standard errors and confidence intervals. This resampling technique helps to overcome the limitations of small sample sizes by creating multiple samples from the original data, thereby providing more reliable statistical inferences. This analysis elucidated the factors affecting drain volume.

## Results

Forty abdominoplasties were performed equally distributed between Groups A (*N* = 20) and B (*N* = 20). The patient characteristics are summarized in Table 1. There were no differences between groups except the BMI. The mean BMI in Group A was significantly higher (*P* = 0.015) than Group B's (25.9 vs 24.2 kg/m^2^). However, it is relevant to note that the weight of the surgical specimen, another important predictor of complications in abdominoplasty, was homogenous between the groups. None of the patients had previously undergone bariatric surgery.

**Table 1:** General characteristics of both groups (*N* = 40)

Characteristic	Traditional `bovie` tip, *N* = 20^1^	HPES tip, *N* = 20^1^	*p*-value^2^
Age (years)			0.4
Mean ± SD	41.4 ± 9.5	43.4 ± 8.3	
[Minimum; Maximum]	[30.0; 61.0]	[32.0; 58.0]	
BMI (kg/m^2^)			**0.015**
Mean ±SD	25.9 ± 2.2	24.2 ± 2.0	
[Minimum; Maximum]	[21.1; 28.5]	[19.4; 27.5]	
Previous abdominal operations	11.0 (55.0%)	11.0 (55.0%)	>0.9
Medical comorbidities	11.0 (55.0%)	10.0 (50.0%)	0.8
Weight specimen (g)			>0.9
Mean ±SD	886.5 ± 354.6	930.7 ± 394.9	
[Minimum; Maximum]	[270.0; 1,500.0]	[344.0; 1,950.0]	

Bold value indicates the statistically significant difference

^1^n (%)

^2^Wilcoxon rank sum test; Fisher's exact test; Pearson's Chi-squared test

*HPES* high-precision electrosurgical scalpel, *BMI* body mass index, *SD* standard deviation

**Fig. 2:**
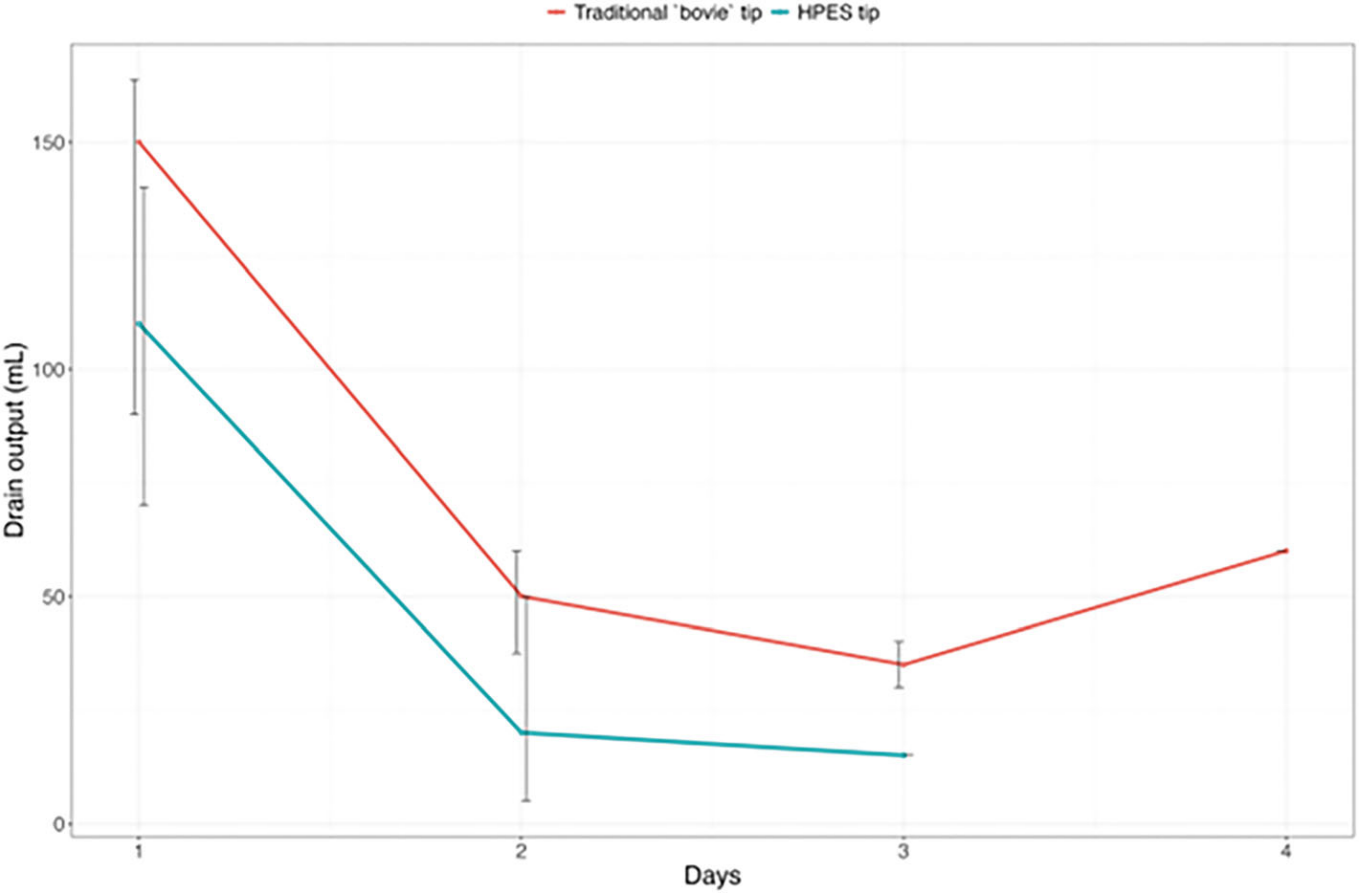
Median daily drain output (mL) along with error bars depicting the 25 and 75 percentiles. The drain output was significantly lower in the HPES tip group on postoperative day 2 (*P* = 0.045, Table 2). HPES: high-precision electrosurgical scalpel

The outcomes are summarized in Table 2. The total drain output presented a statistically significant difference between Groups A and B (*P* = 0.045), corresponding to a 23.6% reduction in Group B. The daily drain output is shown in Fig. 2. There was a trend for lower daily drain outputs in Group B, with reductions of 26.7% on day 1, 60.0% on day 2, and 57.1% on day 3. The drain output of Group B on the 2nd day with drains was significantly lower (*P* = 0.045).

**Table 2:** Outcomes in both groups (*N* = 40).

Outcome	Traditional `bovie` tip, *N* = 20^1^	HPES tip, *N* = 20^1^	*p*-value^2^
Time until drain removal, days			0.3
1	0.0 (0.0%)	1.0 (5.0%)	
2	15.0 (75.0%)	18.0 (90.0%)	
3	4.0 (20.0%)	1.0 (5.0%)	
4	1.0 (5.0%)	0.0 (0.0%)	
Total drain output, mL			**0.045**
Median (IQR)	180.0 (143.8, 287.5)	137.5 (93.8, 185.0)	
[Minimum; Maximum]	[70.0; 390.0]	[40.0; 330.0]	
Drain output on day 1, mL			0.2
Median (IQR)	150.0 (90.0, 163.8)	110.0 (70.0, 140.0)	
[Minimum; Maximum]	[25.0; 290.0]	[40.0; 240.0]	
Drain output on day 2, mL			**0.045**
Median (IQR)	50.0 (37.5, 60.0)	20.0 (5.0, 50.0)	
[Minimum; Maximum]	[0.0; 130.0]	[0.0; 120.0]	
Unknown	0	1	
Drain output on day 3, mL			0.3
Median (IQR)	35.0 (30.0, 40.0)	15.0 (15.0, 15.0)	
[Minimum; Maximum]	[20.0; 120.0]	[15.0; 15.0]	
Unknown	15	19	
Drain output on day 4, mL			
Median (IQR)	60.0 (60.0, 60.0)	–	–
[Minimum; Maximum]	[60.0; 60.0]	–	–
Unknown	19	20	
Emergency dep. visit, n (%)	0 (0%)	0 (0%)	–
Readmission, n (%)	2 (10%)	0 (0%)	0.5
Reoperation, n (%)	2 (10%)	0 (0%)	0.5

Bold values indicate the statistically significant difference

^1^n (%)

^2^Fisher's exact test; Wilcoxon rank sum test; Wilcoxon rank sum exact test

*HPES* high-precision electrosurgical scalpel

No differences were observed between the two groups regarding the time to drain removal. Nevertheless, in Group A, one patient had to use drains for 4 days and four patients for 3 days. In contrast, in Group B, only one patient had to use drains for 3 days, and none required drains for 4 days. There was a trend toward shorter periods of drain use in Group B, as 95 % of the patients in this group had drains removed on the second day, as opposed to 75 % in Group A.

The data in Table 3 presents the complications experienced by both groups. There were no statistically significant differences between the two groups concerning local complications. However, it is worth noting that there was a trend toward a higher incidence of wound dehiscence in Group A.

**Table 3:** Outcomes: systemic and local complications of both groups (*N* = 40)

Outcome	Traditional `bovie` tip, *N* = 20^1^	HPES tip, *N* = 20^1^	*p*-value^2^
Seroma	2 (10%)	2 (10%)	>0.9
Hematoma	1 (5%)	0 (0%)	>0.9
Pseudocapsule	1 (5%)	0 (0%)	>0.9
Wound dehiscence	4 (20%)	2 (10%)	0.7
Infection	0 (0%)	0 (0%)	
Necrose	0 (0%)	0 (0%)	
DVT/PE	0 (0%)	0 (0%)	
Death	0 (0%)	0 (0%)	

^1^n (%)

^2^Fisher's exact test

*HPES* high-precision electrosurgical scalpel, *DVP/PE* deep vein thrombosis/pulmonary embolism

No systemic complications or emergency department visits were recorded for either group. Group A had two cases of readmission and reoperation attributed to wound dehiscence and the development of a pseudo-bursa.

**Fig. 3:**
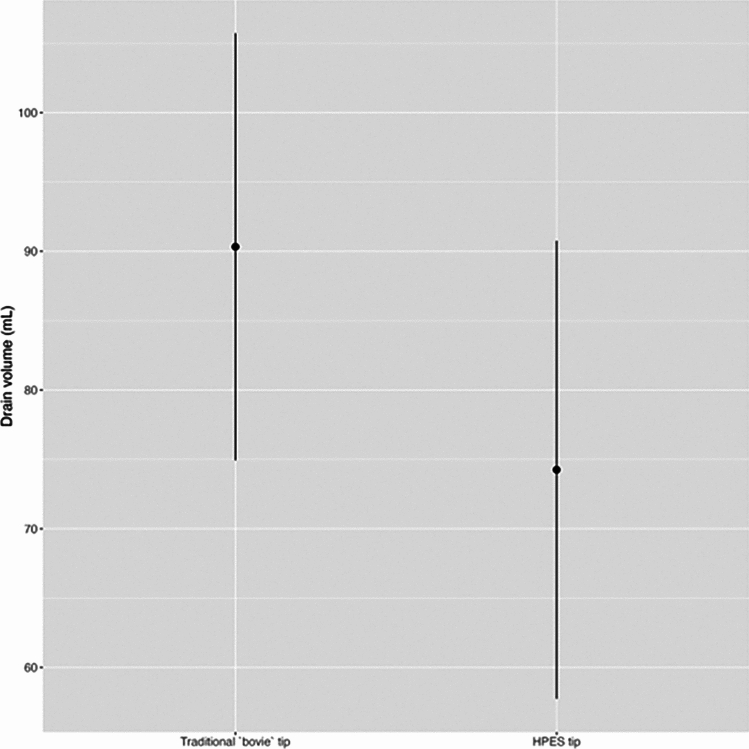
Marginal prediction plot of the average impact of the scalpel on total drain volume (mL). HPES: high-precision electrosurgical scalpel

Moreover, the association between the daily drain volume adjusted for age, BMI, scalpel type, and number of days with drains was examined. The marginal effects plot in Fig. 3 illustrates how the expected drain volume changes concerning the type of electroscalpel while holding other variables constant. This plot suggests that the HPES scalpel was associated with a lower drain volume.

**Fig. 4:**
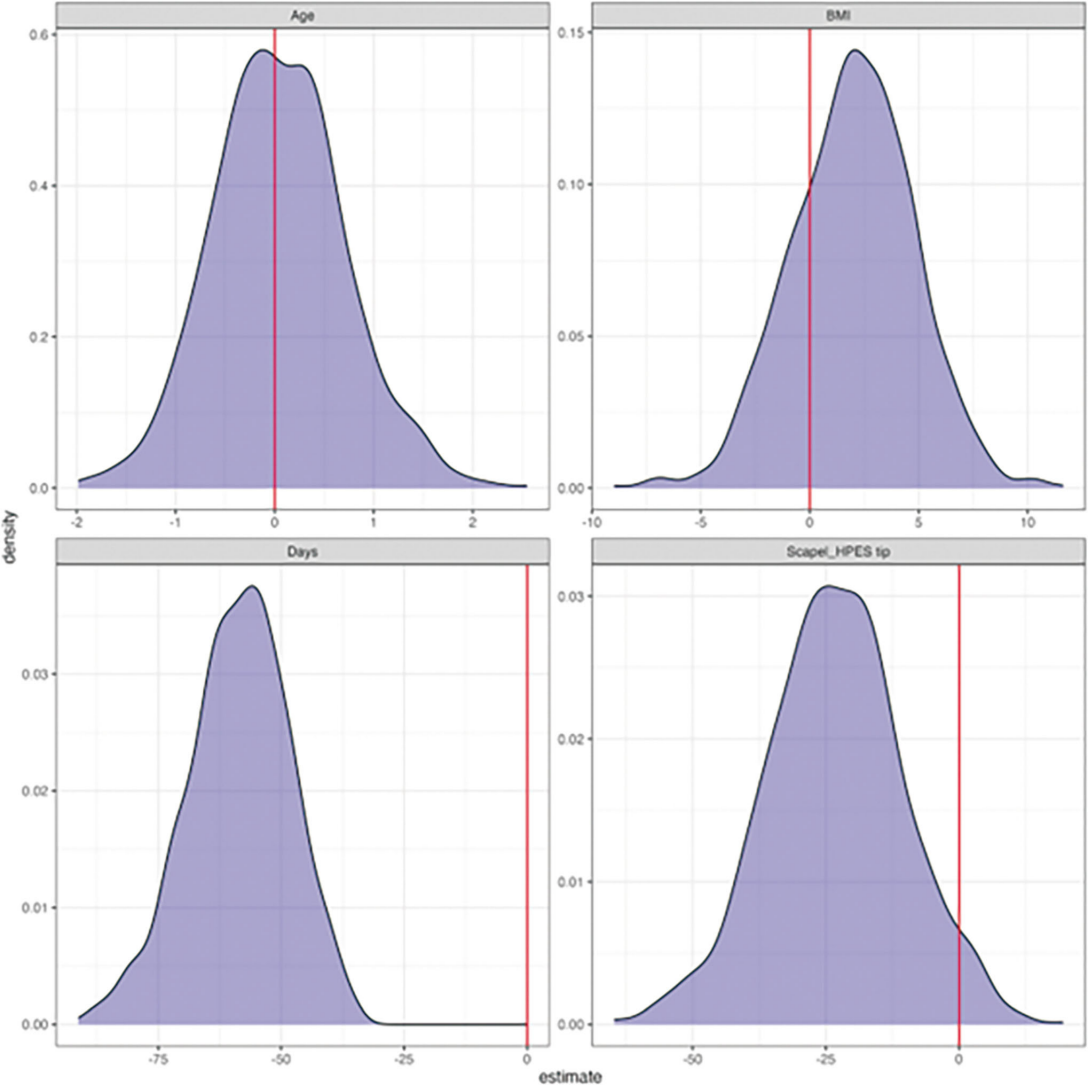
Bootstrap results (1000 runs) of the pre-specified model, daily drain volume (mL) as a function of age, BMI, duration of drain use (days), and scalpel type. Bootstrap analysis of 1000 regression model runs revealed that factors such as BMI, scalpel type, and duration of drain use significantly impact drain volume. These results indicate that the HPES tip is associated with a lower drain volume, whereas a higher BMI is associated with a higher drain volume. Age did not significantly affect drain volume. The reference point, represented by the red line on the zero axis, indicates that there is no effect. HPES: high-precision electrosurgical scalpel

Table 4 presents the relationship between daily drain volume and pre-specified predictors, age, BMI, and scalpel. The regression model shows a negative coefficient for the HPES scalpel predictor, supporting the idea that using the HPES scalpel leads to a lower drain volume, all else being equal. The robustness of this finding was further confirmed by assessing the performance of the HPES scalpel by running 1000 bootstraps models on a pre-specified regression model. The results presented in Fig. 4 demonstrate that employing the HPES scalpel leads to a reduced drainage volume compared with the conventional scalpel.

**Table 4:** Relationship between daily volume drain and pre-specified predictors

Characteristic	Beta	95% CI	p-value
Age	0.10	−1.3, 1.5	0.9
BMI	2.0	−3.7, 7.7	0.5
Scapel			
Traditional ‘bovie’ tip	–	–	
HPES tip	−24	−50, 1.7	0.067
Days	−58	−75, −40	**<0.001**
R^2^	0.363		

Bold value indicates the statistically significant difference

*CI* confidence interval, *BMI* body mass index, *HPES* high-precision electrosurgical scalpel, *R*^*2*^ coefficient of determination

Acknowledging the subjective feedback from the surgeon who favored the HPES over the conventional tip owing to clean and precise tissue dissection is essential. It was evident that HPES easily dissected the adipose tissue without using increased power settings.

## Discussion

Recent publications comparing different dissection techniques to perform an abdominoplasty have not demonstrated a significant advantage of one method over another [15–21], leaving the surgeon’s discretion. This pilot randomized controlled trial provides evidence that Scarpa fascia preservation abdominoplasty using the HPES tip achieved meaningful clinical benefits compared with the conventional tip, with a notable reduction in drain output volumes and improved surgical precision. Specifically, there was a 24% reduction in the total volume of drain output, a significant 60% decrease in drain volume output on the 2nd postoperative day, a tendency toward reduced daily drain volumes, and a tendency toward reduced time of suction drain use. Despite these differences in drainage parameters, similar outcomes and complications were observed with both dissection techniques.

The HPES presents an innovative design that delivers electrical energy to the incision site precisely and selectively while protecting adjacent tissues from dissipated thermal injury [25]. The surgeon involved in this study confirmed the precision and possibility of adipose tissue dissection without needing high-power settings. This is relevant as it has been previously shown that using diathermocoagulation (coagulation mode) regulated aiming at minimal tissue damage reduces drain volume and patients’ time with drains [31]. Nevertheless, a porcine model study by Vore [25] comparing skin incisions made by conventional electrosurgery, HPES, and the steel scalpel for wound healing through histological analysis showed that the steel scalpel significantly favored wound healing when compared to traditional electrosurgery. However, no significant differences were found between the steel scalpel incisions and those made using the HPES. This suggests that HPES may result in postoperative wound healing characteristics closer to the ones produced by the steel scalpel while still providing the benefits associated with electrocautery regarding hemorrhage control [25]. Our results follow Vore’s, as the HPES tip has been shown to reduce the amount of fluid produced, probably because of less tissue trauma.

Surgery relies on the hemostatic and dissection capabilities of conventional electrosurgical devices. Electrocautery was the first energy-based dissection device to be used in surgery, and cauterization is the most common application of this technology [32]. Nevertheless, the underlying mechanism of action, which depends on increasing the temperature of the tissue to cut it, leads to thermal damage to the tissue and its surroundings [33]. Consequently, this triggers an inflammatory cascade, increasing the production of inflammatory cytokines [22] and resulting in higher fluid accumulation and, later, higher seroma formation [17, 18]. Thus, the ideal dissection technique should combine precision with hemostatic control while minimizing associated complications [18]. As detailed in Table 5, prior comparative studies have thoroughly explored dissection techniques, remarkably comparing electrosurgery with new alternative methods. Previously published studies compared steel scalpels with conventional electrosurgery. Marsh [19] compared steel scalpel dissection with electrosurgery using coagulation mode and found no statistically significant differences in the time to drain removal or in seroma incidence. Conversely, Valença-Filipe [18] and Rousseau [17] reported favorable results with a steel scalpel, demonstrating reductions in drain output, time of suction drain use, and a lower incidence of seromas. In a comparative study involving massive weight loss patients after bariatric procedures, Araco [16] found superior results with electrosurgery (without specifying the mode used, i.e., cutting or coagulation) than with a steel scalpel, noting only a statistically significant increase in hematoma incidence in the steel scalpel group. Studies by Duscher [20] and Schlosshauer [21] compared conventional electrosurgery with newer energy-based devices designed to reduce tissue thermal damage but did not demonstrate superiority over traditional electrosurgery. In Duscher’s study, electrocautery was used in the coagulation mode, whereas Schlosshauer employed the cutting mode. Torres-Silva [31] compared the avulsion technique with conventional electrosurgery in coagulation mode and reported a statistically significant reduction in total drain volume and a one-day earlier drain removal with electrocautery. A subgroup analysis of the Torres-Silva investigation demonstrated the advantages of adequately setting the electrosurgical device to minimize thermal damage.

**Table 5:** Studies on dissection technique in abdominoplasty

	Valença-Filipe et al [18]	Marsh et al [19]	Araco et al [16]	Rousseau et al [17]	Duscher et al [20]	Schlosshauer et al [21]	Torres-Silva et al [33]
Type of study	Prospective	Prospective	Retrospective	Retrospective	Prospective	Retrospective	Retrospective
Total no. of patients	119	102	137	647	57	52	251
No. patients electrocautery	80	58	90	327	14	26	129
Electrocautery mode	Coagulation	Coagulation	NA	Cutting	Coagulation	Cutting	Coagulation
No. patients other dissection methods	39	44	47	320	43	26	122
Other dissection method	Scalpel	Scalpel	Scalpel	Scalpel	Energy-based dissection techniques†	PEAK-Plasmablade	Vásconez’s avulsion technique
Drain volume reduction	55% decrease with scalpel	NS	NA	22% decrease with scalpel	NS	NS	Decrease in days 2 to 5 with electrocautery
Earlier drain removal	2d with scalpel	NA	NA	1d with scalpel‡	NA	NS	One day with electrocautery
Seroma	81% decrease with scalpel	NS	NS	Decrease with scalpel	NS	NS	NS
Hematoma/bleeding	NS	NA	Decrease with electrocautery	Decrease with scalpel	Increase with Ultracision	NS	NS
Wound healing problems	90% decrease with scalpel	NA	NS	NS	NS	NS	NS

^†^ PEAK-Plasmablade, Ultracision Harmonic Scalpel, and argon plasma coagulation

^‡^ The criterion for drain removal was 15mL/24-hour period per drain, different from the present study

*NA* not available, *NS* not significant (*P* > 0.05)

Current knowledge suggests that a dissection method superior to conventional electrosurgery has not yet been developed. Although new energy-based methods theoretically have the potential to outperform conventional electrosurgery based on their mechanisms of action, their clinical and economic justification is currently lacking. Therefore, the focus should shift toward strategies aimed at maximizing the efficiency of electrosurgery. This includes meticulous fine-tuning of regulation parameters, as Torres-Silva [31] has shown, and additionally exploring the utilization of specialized tip blades, such as the one used in the current study. Our pilot randomized controlled trial results suggest that HPES may be another strategy to maximize conventional electrosurgery performance with minimal cost, no learning curve, and no need to invest in a new device.

Both dissection methods demonstrated short periods of suction drain use and a low incidence of complications. All abdominoplasties were performed with Scarpa fascia preservation, presenting scientific evidence regarding clinical efficiency [9, 15, 34–37]. A previously published level 1 study [7] highlighted a significant advantage of this technique compared to the classical procedure, revealing a 66% reduction in total drain output and a 47% decrease in time to drain removal. The latter resulted in two crucial clinical advantages, i.e., average suction drain removal 3 days earlier using consensual volumetric criteria and elimination of long periods with suction drains (longer than 6 days). Acknowledging a remarkable shift from 33% to 1% over long periods with suction drains, lasting more than 6 days, is also essential. Moreover, Scarpa sparing resulted in an 87 % reduction in the seroma rate and an 80 % decrease in the hematoma rate. These significant clinical benefits of the Scarpa-sparing technique have also been reported for bariatric patients, a well-known high-risk group for body contour procedures [38–40]. There are two possible explanations for these clinical advantages: physiological and mechanical. By preserving the Scarpa fascia and the thin, deep fat compartment [41, 42] in the infraumbilical area, the lymphatic drainage and vascularization of the anterior abdominal wall are less disturbed [43–46]. On the other hand, preserving the highly flexible deep fat compartment may result in better and faster adhesion between surfaces [47–50].

It is also important to acknowledge the perception of the surgeons involved in the smoother tissue dissection achievable with HPES along with high precision without compromising the duration of surgery. The wider edge of the HPES may have contributed to superior tissue handling.

The HPES is a disposable tip that is attached to the handle of the electroscapel and has the additional advantage of being compatible with different devices. It is possible to incorporate it into the surgeon’s practice while maintaining the same electrosurgical unit, thus minimizing the associated cost of replacing the entire unit. This represents a straightforward and cost-effective strategy to enhance the efficacy and performance of electrosurgical devices.

This study is the first to compare the conventional tip and HPES as dissection methods for Scarpa fascia preservation abdominoplasty. As a pilot study, our analysis established preliminary evidence of the clinical efficacy of HPES while generating essential data for future power calculations. Although the groups showed a statistically significant difference in BMI, the magnitude of this difference was not clinically meaningful, particularly considering that abdominoplasty complications typically correlate with more considerable variations in BMI. This interpretation does not contradict the well-known influence of patient body mass index on abdominoplasty complication rates, usually manifested in higher magnitude BMI differences [51–56]. Another point to consider in our study groups was the weight of the surgical specimens, which was identical. From this point of view, the groups were homogeneous. This is an important point, as previous studies have reported a strong positive correlation between the amount of tissue removed and complications after a full abdominoplasty [54, 57]. One of these studies concluded that the most critical risk factor for seroma is the weight of the surgical specimen [57]. Considering these data, our randomization process achieved balanced groups suitable for meaningful comparisons.

Ongoing efforts should prioritize validation studies to enhance the safety of abdominoplasty. Further research is needed to determine the best dissection method and ideal parameters for electrocautery or other energy-based dissection techniques. The goal is to focus on dissection and undermining procedures that cause the least possible damage.

## Conclusion

This pilot randomized controlled trial suggests that using an HPES tip for tissue dissection in Scarpa-sparing abdominoplasty offers advantages. Specifically, it reduces total drain output by 24%, showing trends toward lower daily drain outputs, shorter periods of drain use, and decreased wound dehiscence. No systemic complications were observed in either group. These findings suggest that the HPES tip is a promising tool for Scarpa-sparing abdominoplasty, warranting further investigation through adequately powered trials to confirm these preliminary results and assess broader surgical applications.

## References

[1] Costa-Ferreira A, Rebelo M, Vásconez L, Amarante J. Scarpa fascia preservation during abdominoplasty. In: Shiffman MA, Di Giuseppe A, editor. Aesthetic plastic surgery of the abdomen. Cham: Springer International Publishing; 2016. p. 59–73.

[2] Pollock TA, Pollock H. Progressive tension sutures in abdominoplasty: a review of 597 consecutive cases. Aesthet Surg J. 2012;32:729–42. 22751080 10.1177/1090820X12452294

[3] Pollock H, Pollock T. Progressive tension sutures: a technique to reduce local complications in abdominoplasty. Plast Reconstr Surg. 2000;105:2583–6. 10845315 10.1097/00006534-200006000-00047

[4] Rao G, Daneshi K, Ceccaroni A, Gentile A, El-Shazali H, Owens N, et al. A systematic review and meta-analysis evaluating the surgical outcomes of progressive tension suturing compared to drains in abdominoplasty surgery. Aesthet Surg J. 2024. 10.1093/asj/sjae171. 39078654 10.1093/asj/sjae171 PMC11634385

[5] Ardehali B, Fiorentino F. A meta-analysis of the effects of abdominoplasty modifications on the incidence of postoperative seroma. Aesthet Surg J. 2017;37:1136–43. 28482000 10.1093/asj/sjx051

[6] Costa-Ferreira A, Rebelo M, Vasconez LO, Amarante J. Scarpa fascia preservation during abdominoplasty: a prospective study. Plast Reconstr Surg. 2010;125:1232–9. 20072084 10.1097/PRS.0b013e3181d0ac59

[7] Costa-Ferreira A, Rebelo M, Silva A, Vásconez LO, Amarante J. Scarpa fascia preservation during abdominoplasty: randomized clinical study of efficacy and safety. Plast Reconstr Surg. 2013;131:644–51. 23446574 10.1097/PRS.0b013e31827c704b

[8] Novais CS, Carvalho J, Valenca-Filipe R, Rebelo M, Peres H, Costa-Ferreira A. Abdominoplasty with Scarpa fascia preservation: randomized controlled trial with assessment of scar quality and cutaneous sensibility. Plast Reconstr Surg. 2020;146:156e–64e. 32740578 10.1097/PRS.0000000000007024

[9] van der Sluis N, van Dongen JA, Caris FLS, Wehrens KME, Carrara M, van der Lei B. Does Scarpa’s fascia preservation in abdominoplasty reduce seroma? A systematic review. Aesthet Surg J. 2023. 10.1093/asj/sjad024. 36747469 10.1093/asj/sjad024 PMC10264224

[10] Saldanha OR, Federico R, Daher PF, Malheiros AA, Carneiro PRG, Azevedo SFD, et al. Lipoabdominoplasty. Plast Reconstr Surg. 2009;124:934–42. 19730314 10.1097/PRS.0b013e3181b037e3

[11] Saldanha OR, De Souza Pinto EB, Mattos WN Jr., Pazetti CE, Lopes Bello EM, Rojas Y, et al. Lipoabdominoplasty with selective and safe undermining. Aesthetic Plast Surg. 2003;27:322–7. 15058559 10.1007/s00266-003-3016-z

[12] Andrades P, Prado A, Danilla S, Guerra C, Benitez S, Sepulveda S, et al. Progressive tension sutures in the prevention of postabdominoplasty seroma: a prospective, randomized, double-blind clinical trial. Plast Reconstr Surg. 2007;120:935–46. 17805122 10.1097/01.prs.0000253445.76991.de

[13] Pisco A, Rebelo M, Peres H, Costa-Ferreira A. Abdominoplasty with Scarpa fascia preservation: prospective comparative study of suction drain number. Ann Plast Surg. 2020;84:356–60. 32149854 10.1097/SAP.0000000000002349

[14] Khansa I, Khansa L, Meyerson J, Janis JE. Optimal use of surgical drains: evidence-based strategies. Plast Reconstr Surg. 2018;141:1542–9. 29608530 10.1097/PRS.0000000000004413

[15] Janis JE, Khansa L, Khansa I. Strategies for postoperative seroma prevention: a systematic review. Plast Reconstr Surg. 2016;138:240–52. 27348657 10.1097/PRS.0000000000002245

[16] Araco A, Sorge R, Overton J, Araco F, Gravante G. Postbariatric patients undergoing body-contouring abdominoplasty: two techniques to raise the flap and their influence on postoperative complications. Ann Plast Surg. 2009;62:613–7. 19461270 10.1097/SAP.0b013e3181856d85

[17] Rousseau P, Vincent H, Potier B, Arnaud D, Darsonval V. Diathermocoagulation in cutting mode and large flap dissection. Plast Reconstr Surg. 2011;127:2093–8. 21532437 10.1097/PRS.0b013e31820cf46e

[18] Valenca-Filipe R, Martins A, Silva A, Vasconez LO, Amarante J, Costa-Ferreira A. Dissection technique for abdominoplasty: a prospective study on scalpel versus diathermocoagulation (coagulation mode). Plast Reconstr Surg Glob Open. 2015;3:e299. 25674380 10.1097/GOX.0000000000000222 PMC4323403

[19] Marsh DJ, Fox A, Grobbelaar AO, Chana JS. Abdominoplasty and seroma: a prospective randomised study comparing scalpel and handheld electrocautery dissection. J Plast Reconstr Aesthet Surg. 2015;68:192–6. 25456290 10.1016/j.bjps.2014.10.004

[20] Duscher D, Aitzetmuller MM, Shan JJ, Wenny R, Brett EA, Staud CJ, et al. Comparison of energy-based tissue dissection techniques in abdominoplasty: a randomized, open-label study including economic aspects. Aesthet Surg J. 2019;39:536–43. 30016404 10.1093/asj/sjy177

[21] Schlosshauer T, Kiehlmann M, Riener MO, Sader R, Rieger UM. Comparative analysis on the effect of low-thermal plasma dissection device (PEAK PlasmaBlade) vs conventional electrosurgery in post-bariatric abdominoplasty: a retrospective randomised clinical study. Int Wound J. 2019;16:1494–502. 31531963 10.1111/iwj.13221 PMC7949000

[22] Litta P, Saccardi C, Gizzo S, Conte L, Ambrosi G, Sissi C, et al. Inflammatory cytokine expression following the use of bipolar electrocoagulation, ultracision harmonic scalpel and cold knife biopsy. Mol Med Rep. 2015;12:2985–90. 25937018 10.3892/mmr.2015.3677

[23] Andrades P, Prado A. Composition of postabdominoplasty seroma. Aesthet Plast Surg. 2007;31:514–8. 10.1007/s00266-007-0078-3 17659406

[24] Chaouat M, Levan P, Lalanne B, Buisson T, Nicolau P, Mimoun M. Abdominal dermolipectomies: early postoperative complications and long-term unfavorable results. Plast Reconstr Surg. 2000;106:1614–8. 11129195 10.1097/00006534-200012000-00029

[25] Vore SJ, Wooden WA, Bradfield JF, Aycock ED, Vore PL, Lalikos JF, et al. Comparative healing of surgical incisions created by a standard “bovie,” the Utah Medical Epitome Electrode, and a Bard-Parker cold scalpel blade in a porcine model: a pilot study. Ann Plast Surg. 2002;49:635–45. 12461448 10.1097/00000637-200212000-00014

[26] Matarasso A, Matarasso DM, Matarasso EJ. Abdominoplasty: classic principles and technique. Clin Plast Surg. 2014;41:655–72. 25283453 10.1016/j.cps.2014.07.005

[27] Bozola AR. Abdominoplasty: same classification and a new treatment concept 20 years later. Aesthet Plast Surg. 2010;34:181–92. 10.1007/s00266-009-9407-z 19768494

[28] Costa-Ferreira A, Marco R, Vasconez L, Amarante J. Abdominoplasty with Scarpa fascia preservation. Ann Plast Surg. 2016;76(Suppl 4):S264-274. 27187249 10.1097/SAP.0000000000000851

[29] The R Foundation for Statistical Computing. R: A language and environment for statistical computing. 2023. https://www.R-project.org/.

[30] Sjoberg DDWK, Curry M, Lavery JA, Larmarange J. Reproducible summary tableswith the gtsummary package. The R J. 2021;13:570–80.

[31] Torres-Silva C, Pisco A, Valenca-Filipe R, Rebelo M, Peres H, Vasconez L, et al. Dissection technique for abdominoplasty with Scarpa fascia preservation: comparative study on avulsion technique versus diathermocoagulation. Aesthet Surg J. 2021. 10.1093/asj/sjaa428. 33403390 10.1093/asj/sjaa428

[32] Massarweh NN, Cosgriff N, Slakey DP. Electrosurgery: history, principles, and current and future uses. J Am Coll Surg. 2006;202:520–30. 16500257 10.1016/j.jamcollsurg.2005.11.017

[33] Ruidiaz ME, Messmer D, Atmodjo DY, Vose JG, Huang EJ, Kummel AC, et al. Comparative healing of human cutaneous surgical incisions created by the PEAK PlasmaBlade, conventional electrosurgery, and a standard scalpel. Plast Reconstr Surg. 2011;128:104–11. 21701326 10.1097/PRS.0b013e31821741ed

[34] Wijaya WA, Liu Y, He Y, Qing Y, Li Z. Abdominoplasty with Scarpa fascia preservation: a systematic review and meta-analysis. Aesthetic Plast Surg. 2022. 10.1007/s00266-022-02835-5. 35301571 10.1007/s00266-022-02835-5

[35] Xiao X, Ye L. Efficacy and safety of Scarpa fascia preservation during abdominoplasty: a systematic review and meta-analysis. Aesthetic Plast Surg. 2017. 10.1007/s00266-017-0784-4. 28405750 10.1007/s00266-017-0784-4

[36] Seretis K, Goulis D, Demiri EC, Lykoudis EG. Prevention of seroma formation following abdominoplasty: a systematic review and meta-analysis. Aesthet Surg J. 2017;37:316–23. 28158391 10.1093/asj/sjw192

[37] Ardehali B, Fiorentino F. A meta-analysis of the effects of abdominoplasty modifications on the incidence of postoperative seroma. Aesthet Surg J. 2017. 10.1093/asj/sjx051. 28482000 10.1093/asj/sjx051

[38] Correia-Goncalves I, Valenca-Filipe R, Carvalho J, Rebelo M, Peres H, Amarante J, et al. Abdominoplasty with Scarpa fascia preservation - comparative study in a bariatric population. Surg Obes Relat Dis. 2017;13:423–8. 27889486 10.1016/j.soard.2016.09.024

[39] Monteiro IA, de Sousa Barros A, Costa-Ferreira A. Postbariatric abdominoplasty: a comparative study on Scarpa fascia preservation versus classical technique. Aesthetic Plast Surg. 2023. 10.1007/s00266-023-03455-3. 37365309 10.1007/s00266-023-03455-3

[40] Alves M, Mendes M, Valenca-Filipe R, Rebelo M, Peres H, Costa-Ferreira A. Long drainers after abdominoplasty: a risk analysis. Aesthet Plast Surg. 2025. 10.1007/s00266-025-04773-4. 10.1007/s00266-025-04773-4 PMC12133941 40131399

[41] Costa-Ferreira A, Rodrigues-Pereira P, Rebelo M, Vásconez LO, Amarante J. Morphometric study (macroscopic and microscopic) of the lower abdominal wall. Plast Reconstr Surg. 2014;134:1313–22. 25255112 10.1097/PRS.0000000000000732

[42] Valenca-Filipe R, Mendes J, Pereira F, Vardasca R, Amarante J, Costa-Ferreira A. Physical properties of Scarpa’s fascia. Clin Anat. 2023;37(4):397–404. 37377018 10.1002/ca.24087

[43] Valenca-Filipe R, Vardasca R, Magalhaes C, Mendes J, Amarante J, Costa-Ferreira A. Classic versus Scarpa-sparing abdominoplasty: an infrared thermographic comparative analysis. J Plast Reconstr Aesthet Surg. 2023;82:264–74. 37209599 10.1016/j.bjps.2023.04.023

[44] Friedman T, Coon D, Kanbour-Shakir A, Michaels Jt, Rubin JP. Defining the lymphatic system of the anterior abdominal wall: an anatomical study. Plast Reconstr Surg. 2015;135:1027–32. 25811569 10.1097/PRS.0000000000001136

[45] Albertin G, Astolfi L, Fede C, Simoni E, Contran M, Petrelli L, et al. Detection of lymphatic vessels in the superficial fascia of the abdomen. Life. 2023. 10.3390/life13030836. 36983991 10.3390/life13030836 PMC10058564

[46] Pirri C, Petrelli L, Fede C, Guidolin D, Tiengo C, De Caro R, et al. Blood supply to the superficial fascia of the abdomen: an anatomical study. Clin Anat. 2023;36:570–80. 36576229 10.1002/ca.23993

[47] Lancerotto L, Stecco C, Macchi V, Porzionato A, Stecco A, De Caro R. Layers of the abdominal wall: anatomical investigation of subcutaneous tissue and superficial fascia. Surg Radiol Anat. 2011;33:835–42. 21212951 10.1007/s00276-010-0772-8

[48] Nakajima H, Imanishi N, Minabe T, Kishi K, Aiso S. Anatomical study of subcutaneous adipofascial tissue: a concept of the protective adipofascial system (PAFS) and lubricant adipofascial system (LAFS). Scand J Plast Reconstr Surg Hand Surg. 2004;38:261–6. 15513595 10.1080/02844310410029543

[49] Costa-Ferreira A, Vasconez LO, Amarante J. Reply: Scarpa fascia preservation during abdominoplasty: randomized clinical study of efficacy and safety. Plast Reconstr Surg. 2013;132:873e–4e. 24165648 10.1097/PRS.0b013e3182a4c510

[50] Pisco A, Rebelo M, Peres H, Costa-Ferreira A. Response to comments on abdominoplasty with Scarpa Fascia preservation prospective comparative study of suction drain number. Ann Plast Surg. 2021;86:486–7. 32804720 10.1097/SAP.0000000000002529

[51] Neaman KC, Armstrong SD, Baca ME, Albert M, Vander Woude DL, Renucci JD. Outcomes of traditional cosmetic abdominoplasty in a community setting: a retrospective analysis of 1008 patients. Plast Reconstr Surg. 2013;131:403e–10e. 23446591 10.1097/PRS.0b013e31827c6fc3

[52] Kim J, Stevenson TR. Abdominoplasty, liposuction of the flanks, and obesity: analyzing risk factors for seroma formation. Plast Reconstr Surg. 2006;117:773–9. 16525264 10.1097/01.prs.0000200056.57357.3f

[53] Winocour J, Gupta V, Ramirez JR, Shack RB, Grotting JC, Higdon KK. Abdominoplasty: risk factors, complication rates, and safety of combined procedures. Plast Reconstr Surg. 2015;136:597e–606e. 26505716 10.1097/PRS.0000000000001700

[54] Neaman KC, Hansen JE. Analysis of complications from abdominoplasty: a review of 206 cases at a university hospital. Ann Plast Surg. 2007;58:292–8. 17471135 10.1097/01.sap.0000239806.43438.54

[55] Vastine VL, Morgan RF, Williams GS, Gampper TJ, Drake DB, Knox LK, et al. Wound complications of abdominoplasty in obese patients. Ann Plast Surg. 1999;42:34–9. 9972715 10.1097/00000637-199901000-00006

[56] Rogliani M, Silvi E, Labardi L, Maggiulli F, Cervelli V. Obese and nonobese patients: complications of abdominoplasty. Ann Plast Surg. 2006;57:336–8. 16929206 10.1097/01.sap.0000221460.43861.6b

[57] Shermak MA, Rotellini-Coltvet LA, Chang D. Seroma development following body contouring surgery for massive weight loss: patient risk factors and treatment strategies. Plast Reconstr Surg. 2008;122:280–8. 18594418 10.1097/PRS.0b013e31817742a9

